# Supplementary material for: Metabolomic machine learning predictor for diagnosis and prognosis of gastric cancer
Source: Nat Commun. 2024 Feb 23;15:1657. doi: 10.1038/s41467-024-46043-y (PMC10891053; doi:10.1038/s41467-024-46043-y)
Supplement: Supplementary file 1 — Supplementary Information [file 41467_2024_46043_MOESM1_ESM.pdf]

Metabolomic machine learning predictor for diagnosis and prognosis of gastric cancer

Yangzi Chen[1,12], Bohong Wang[1,2,12], Yizi Zhao[1,12], Xinxin Shao[3,12], Mingshuo Wang[1,2,12], Fuhai Ma[3,4,12], Laishou Yang[5], Meng Nie[1], Peng Jin[3,6], Ke Yao[1], Haibin Song[7], Shenghan Lou[5], Hang Wang[5], Tianshu Yang[8,9], Yantao Tian[3]*, Peng Han[10,11]*, Zeping Hu[1,2]*

[1] School of Pharmaceutical Sciences, Tsinghua University, Beijing, 100084, China.

[2] Tsinghua-Peking Joint Center for Life Sciences, Tsinghua University, Beijing, 100084, China.

[3] National Cancer Center, National Clinical Research Center for Cancer, Cancer Hospital, Chinese Academy of Medical Sciences, Peking Union Medical College, Beijing, 100730, China.

[4] Department of General Surgery, Department of Gastrointestinal Surgery, Beijing Hospital, National Center of Gerontology, Institute of Geriatric Medicine, Chinese Academy of Medical Sciences, Beijing, 100730, China

[5] Department of Colorectal Surgery, Harbin Medical University Cancer Hospital, Harbin, 150081, China.

[6] Department of Gastroenterology, Tianjin Medical University Cancer Institute and Hospital, National Clinical Research Center for Cancer, Key Laboratory of Cancer Prevention and Therapy, Tianjin's Clinical Research Center for Cancer, Tianjin, 300060, China.

[7] Department of Gastrointestinal Surgery, Harbin Medical University Cancer Hospital, Harbin, 150081, China.

[8] Shanghai Key Laboratory of Metabolic Remodeling and Health, Institute of Metabolism and Integrative Biology, Institutes of Biomedical Sciences, Fudan University, Shanghai, 200032, China

[9] Shanghai Qi Zhi Institute, Shanghai, 200438, China

[10] Department of Oncology Surgery, Harbin Medical University Cancer Hospital, Harbin, 150081, China.

[11] Key Laboratory of Tumor Immunology in Heilongjiang, Harbin, 150081, China.

[12] These authors contributed equally

*Correspondence:

tianyantao@cicams.ac.cn (Y.T.); leospiv@hrbmu.edu.cn (P. H.); zeping_hu@tsinghua.edu.cn (Z.H.)

Supplementary Figures

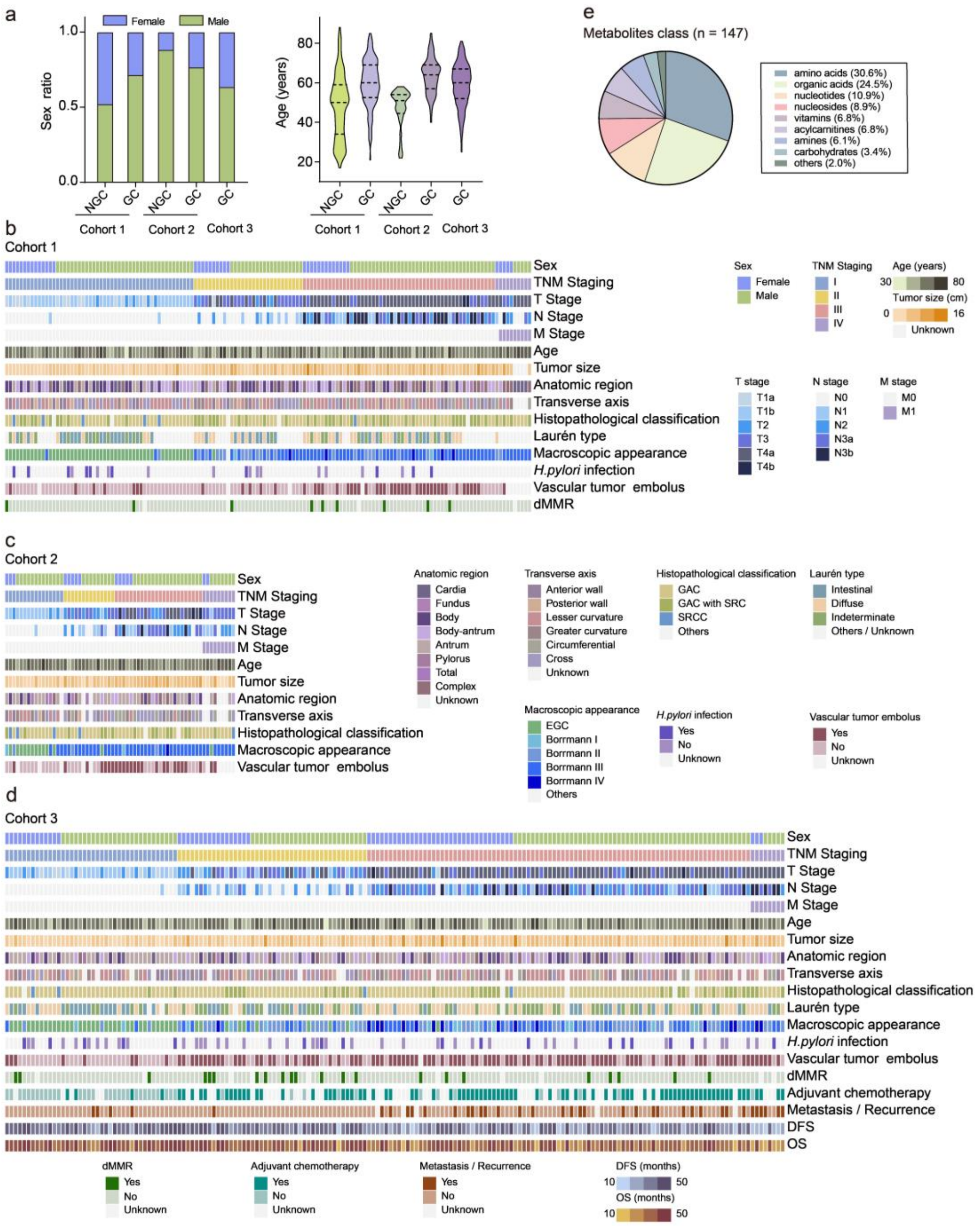

Supplementary Fig. 1 | Participant recruitment and metabolites composition.

a-d, Clinical characteristics of Cohort 1-3 subjects. The dashed lines represent the median and quartiles. **e,** Classes, and proportions of metabolites detected in the study. dMMR: Deficient mismatch repair, GAC: Gastric adenocarcinoma, SRC: Signet ring cell, SRCC: Signet ring cell cancer, EGC: Early gastric cancer. Source data are provided as a Source Data file.

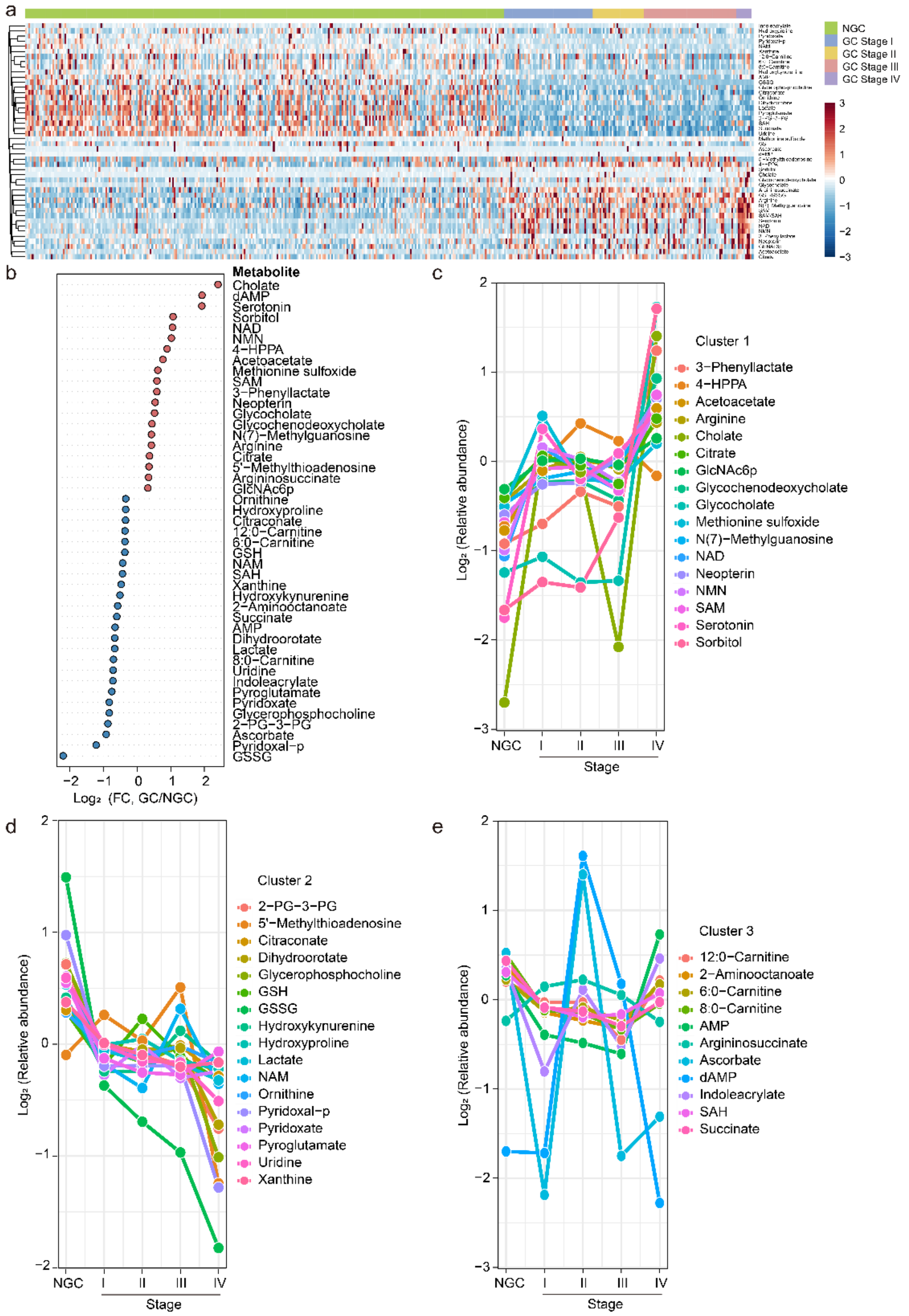

Supplementary Fig. 2 | Metabolomics analysis of GC patients and NGC controls.

a, Heatmap of differential metabolites between GC and NGC in Cohort 1. **b,** The differential metabolites in GC versus NGC. Two-sided Wilcoxon rank-sum tests followed by Benjamini-Hochberg (BH) multiple comparison test with false discovery rate (FDR) < 0.05 and fold change (FC) > 1.25 or <0.8. Up-regulated metabolites in GC were colored in red while down-regulated metabolites were colored in blue. **c-e,**

Dynamic alterations of metabolites in clusters 1-3. The dots represent the mean log2 relative abundance. Source data are provided as a Source Data file.

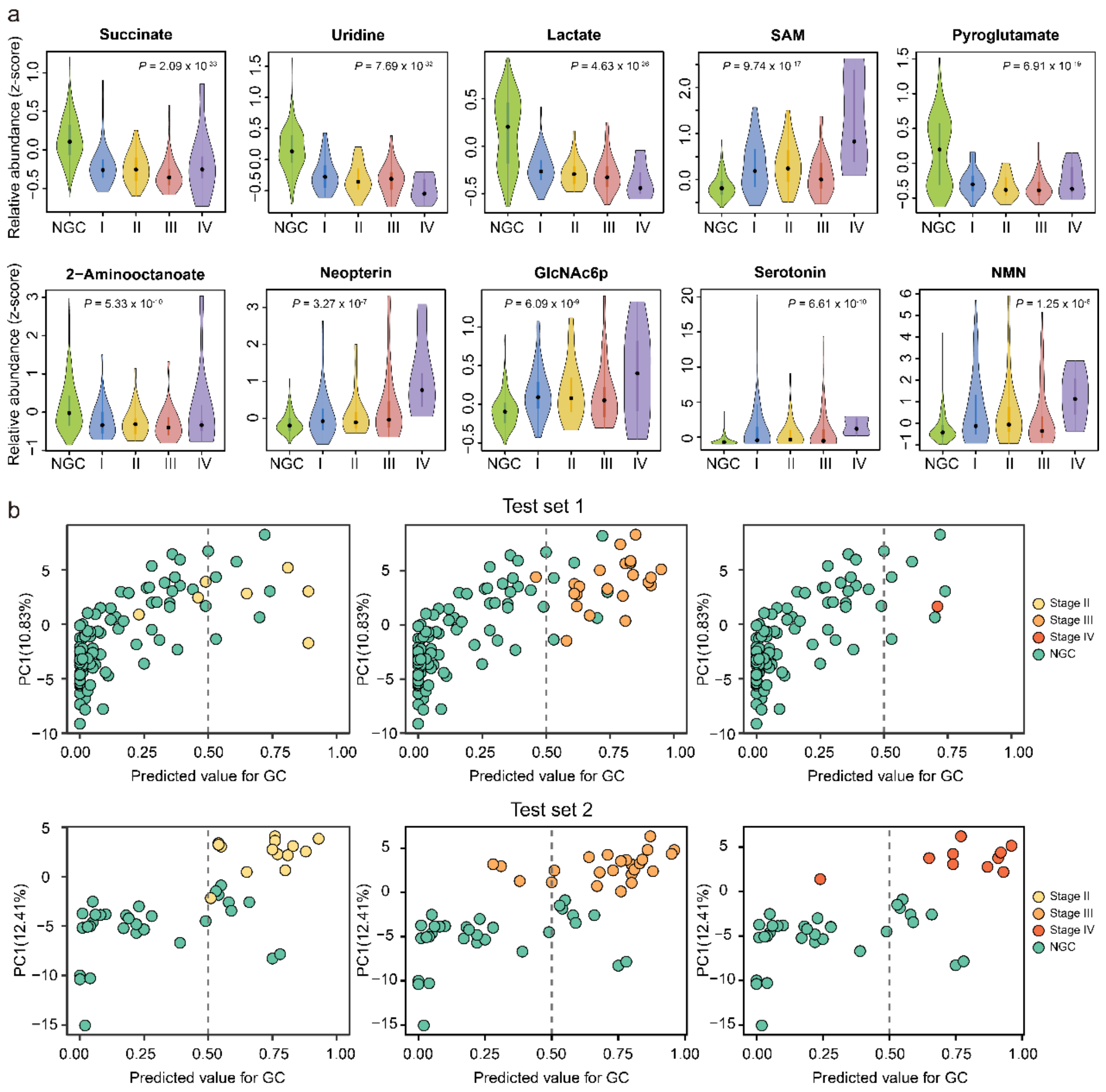

Supplementary Fig. 3 | Metabolite distribution and performance evaluation of the 10-DM model.

a, Violin plots of the modeling metabolites using relative abundance (the z-score transformed original normalized peak area) among Cohort 1, including NGC (n=281) and GC (Stage I, n=52; Stage II, n=30; Stage III, n=53; Stage IV, n=10) plasma samples. The differences were calculated using the two-sided Kruskal-Wallis test. Black dots represent population medians. **b,** The prediction performance of the 10-DM model for distinguishing stage II/III/IV GC (colored in yellow, orange and red) from NGC in test sets 1 and 2. The dotted line represented the cut-off value of 0.50 used to separate the predicted NGC (on the left side) from GC (on the right side).

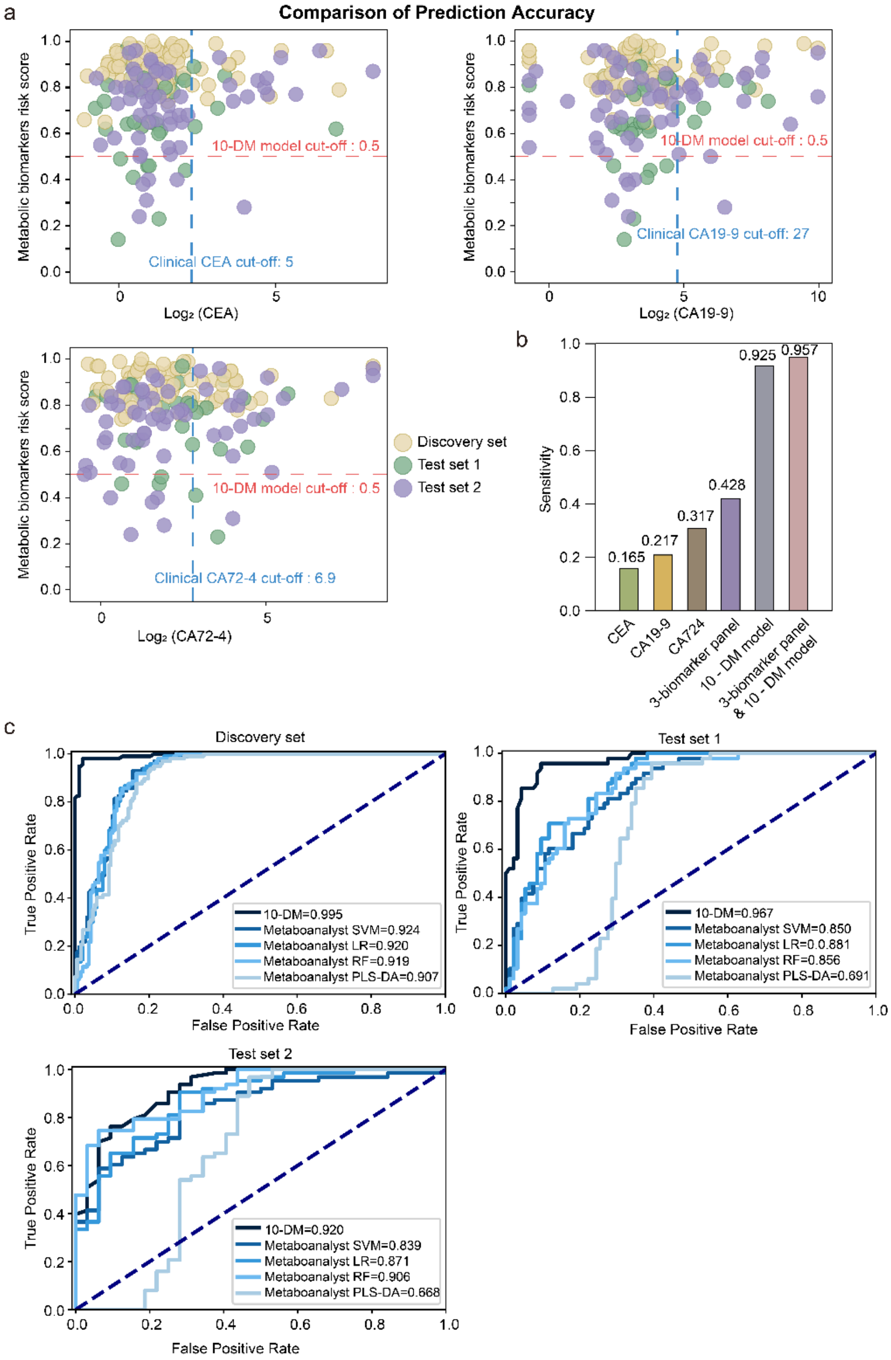

Supplementary Fig. 4 | Diagnostic accuracy comparison of the 10-DM model and clinical markers.

a, Diagnostic prediction of the GC patients using the 10-DM model and clinical markers respectively. The discovery set, test set 1 and 2 GC patients were colored in yellow, green, and purple respectively. The blue dotted line represents the log2 cutoff value of each marker, while the red dotted line represents the cutoff value of the 10-DM model. **b,** Comparison of different markers and models' detection sensitivity in predicting GC patients. CEA, carcinoembryonic antigen; CA19-9, carbohydrate

antigen 19-9; CA724, carbohydrate antigen 724. **c,** The AUROC curves for the 10-DM model and various machine learning models constructed using Metaboanalyst are depicted for the discovery set, test set 1 and test set 2.

a

Weight	Feature	Weight	Feature	Weight	Feature
0.0351±0.0492	SDMA/ADMA	0.0108±0.0105	Indoleacrylate	0.0022±0.0102	Riboflavin
0.0316±0.0250	Neopterin	0.0099±0.0081	8 : 0-Carnitine	0.0021±0.0037	Uracil
0.0243±0.0166	Thymine	0.0090±0.0057	12 : 0-Carnitine	0.0018±0.0036	Dihydroorotate
0.0177±0.0082	Glucuronate	0.0087±0.0045	Acetylalanine	0.0015±0.0028	R5P
0.0155±0.0094	Isocitrate	0.0086±0.0062	2-Aminoadipate	0.0014±0.0186	GlcNAc6p
0.0152±0.0113	Uridine	0.0075±0.0060	Creatinine	-0.0003±0.0013	Serotonin
0.0139±0.0091	Hydroxyproline	0.0046±0.0030	Homocysteine	-0.0046±0.0060	Carnitine
0.0126±0.0040	Succinate	0.0046±0.0049	AICAR	-0.0058±0.0100	Glycochenodeoxycholate
0.0121±0.0066	N-Acetylputrescine	0.0036±0.0023	Acetylaspartate		
0.0120±0.0049	14 : 0- Carnitine	0.0022±0.0066	Cytidine		

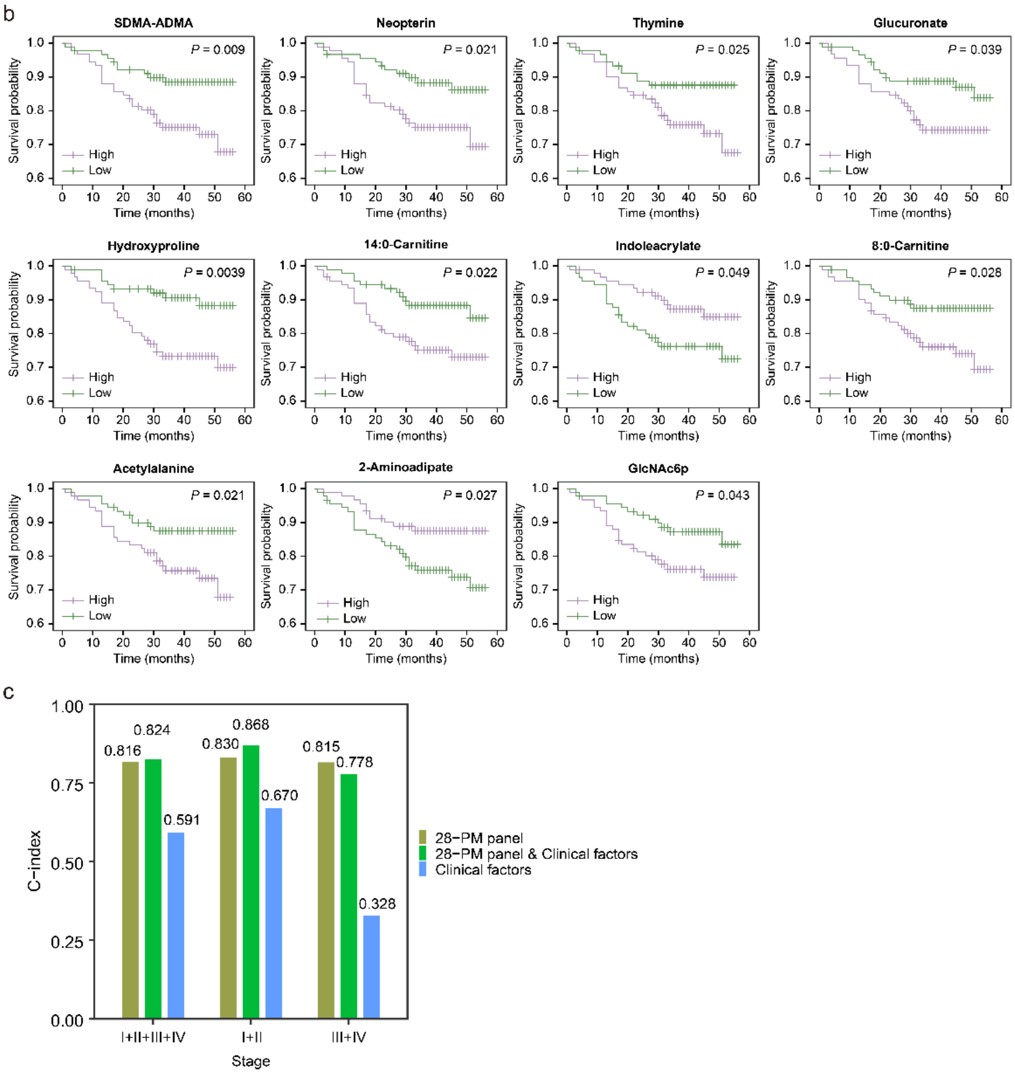

Supplementary Fig. 5 | Characteristics of the 28-PM model.

a, Weights of the 28 metabolic features in the 28-PM model. **b,** Kaplan–Meier curves for the overall survival of test set GC patients stratified by 28-PM model metabolites with a two-sided log-rank test. The patients were divided into high and low groups by the median of the metabolite abundances in GC patients. **c,** C-index comparison of models, including the 28-PM model, the 28-PM panel integrated with clinical factors, and clinical factors, for predicting the prognosis of GC patients at different stages.

Table S1 Characteristics of the clinical parameters that were not significantly associated with GC patients' prognosis. Univariate Cox regression analysis was performed on clinical parameters to identify those with significant prognostic correlations. Parameters with P>0.05 are considered statistically insignificant, indicating a lack of significant association with the prognosis of GC patients. The hazard ratio, 95% CI (Confidence interval) and P value were calculated by univariate Cox regression analysis.GAC, Gastric adenocarcinoma; SRCC, Gastric signet-ring cell carcinoma; dMMR, deficient Mismatch repair.

Characteristics	Classificatior	Frequency	*P* value	Hazard ratio	95% CI
**Sex**	Male	114 (62.98%)		Reference	
	Female	67 (37.02%)	0.621	0.84	0.42 - 1.69
**Age (years)**	<40	10 (5.52%)		Reference	
	41-50	31 (17.13%)	0.918	1.09	0.22 - 5.39
	51-60	54 (29.83%)	0.934	0.94	0.20 - 4.34
	61-70	63 (34.81%)	0.951	0.95	0.21 - 4.30
	>70	23 (12.71%)	0.494	1.73	0.36 - 8.34
**Tumor size (cm)**	<2.5	37 (20.44%)		Reference	
	2.5-5	91 (50.28%)	0.650	1.26	0.46 - 3.48
	>5	53 (29.28%)	0.090	2.40	0.87 - 6.61
**Anatomic region**	Cardia	13 (7.18%)		Reference	
	Fundus	4 (2.21%)	0.787	1.39	0.13 - 15.36
	Body	33 (18.23%)	0.744	1.30	0.27 - 6.26
	Body-antrum	35 (19.34%)	0.679	0.70	0.13 - 3.82
	Antrum	80 (44.2%)	0.934	1.06	0.24 - 4.69
	Pylorus	2 (1.10%)	0.261	3.96	0.36 - 43.87
	Total	2 (1.10%)	0.244	4.17	0.38 - 46.22
	Complex	12 (6.63%)	0.180	3.09	0.60 - 16.01
**Transverse axis**	Anterior wall	16 (9.82%)		Reference	
	Posterior wall	15 (9.20%)	0.543	0.47	0.04 - 5.23
	Lesser curvature	74 (45.40%)	0.488	1.69	0.39 - 7.38
	Greater curvature	15 (9.20%)	0.675	1.47	0.24 - 8.78
	Circumferential	20 (12.27%)	0.367	2.13	0.41 - 10.97
	Cross	23 (14.11%)	0.341	0.31	0.03 -3.43
**Histopathological classification**	GAC	138 (78.41%)		Reference	
	GAC with SRC	34 (19.32%)	0.662	1.19	0.54 - 2.62
	SRCC	4 (2.27%)	0.977	-	-
**Lauren type**	Intestinal	48 (27.43%)		Reference	
	Diffuse	73 (41.71%)	0.205	1.84	0.72 - 4.69
	Indeterminate	54 (30.86%)	0.147	2.05	0.78 - 5.38
***H.pylori* infection**	No	37 (68.52%)		Reference	
	Yes	17 (31.48%)	0.891	1.13	0.21 - 6.20
**dMMR**	No	149 (87.13%)		Reference	
	Yes	22 (12.87%)	0.207	0.40	0.10 - 1.66
**Adjuvant chemotherapy**	No	44 (33.59%)		Reference	
	Yes	87 (66.41%)	0.383	1.51	0.60 - 3.80
