# Supplementary material for: Metabolomic machine learning predictor for diagnosis and prognosis of gastric cancer
Source: Nat Commun. 2024 Feb 23;15:1657. doi: 10.1038/s41467-024-46043-y (PMC10891053; doi:10.1038/s41467-024-46043-y)
Supplement: Supplementary file 4 — Description of Additional Supplementary Files [file 41467_2024_46043_MOESM4_ESM.pdf]

Description of Additional Supplementary Files

Supplementary Table 1: Characteristics of the clinical parameters that were not significantly associated with GC patients' prognosis.

Supplementary Data 1: Metabolomic data utilized for the construction of diagnostic and prognostic models.

Supplementary Data 2: The raw statistical data pertinent to Supplementary Fig.3.

Supplementary Data 3: The methodological evaluation data for the targeted metabolomic approach in this study, comprising matrix effects, recovery rates, quantitative accuracy, and relative quantitative precision.

Supplementary Data 4: The utilization of targeted metabolomics approach for absolute quantification analysis in a small cohort.

Supplementary Data 5: Details on the model's predicted values for all samples.

Supplementary Data 6: The statistical analysis of the proportions of cMET amplification, dMMR, and Laurén types between the high and low risk groups.
